# Mitochondrial transplant: Unravelling a promising treatment for ocular diseases

**DOI:** 10.1016/j.redox.2025.103902

**Published:** 2025-10-17

**Authors:** Yan Zhuang Yeo, Mayuri Bhargava, Rajvinder Singh Khare, Bhav Harshad Parikh, Xinyi Su

**Affiliations:** a Department of Ophthalmology, Yong Loo Lin School of Medicine, National University of Singapore (NUS), 1E Kent Ridge Road, NUHS Tower Block Level 7, Singapore, 119228, Singapore; b Institute of Molecular and Cell Biology (IMCB), Agency for Science, Technology and Research (A∗STAR), 61 Biopolis Drive, Singapore, 138673, Singapore; c Department of Ophthalmology, National University Hospital, 1E Kent Ridge Road, NUHS Tower Block Level 7, Singapore, 119228, Singapore; d Singapore Eye Research Institute, The Academia, 20 College Road, Level 6 Discovery Tower, Singapore, 169856, Singapore

**Keywords:** Mitochondria transplantation, Ocular metabolic diseases, Bioenergetic rescue, Oxidative stress, Mitochondrial biology, Clinical translation

## Abstract

Mitochondrial transplantation is an upcoming therapeutic modality where transfer of healthy robust mitochondria bio-enhances metabolically dysfunctional cells or tissues. Though the concept of MT germinated in early 1980s in a bid to develop antibiotic resistance between cells, this innovative treatment has since undergone various breakthroughs in addressing metabolic dysfunction in various systemic diseases. Four decades since its advent, MT is now being applied in the field of Ophthalmology, where metabolic disorders affecting various ocular tissues contribute significantly to disease pathogenesis. Encouraged by the success of MT in other organs such as heart, lung and brain, this therapy has recently been applied to ocular disorders. MT is an emerging ocular therapy, with promising therapeutic outcomes for corneal, optic nerve, and retinal disorders. However, before it can be adopted as a “bench to bedside” therapy for ocular disorders, MT faces several potential bottlenecks. This review provides an overview of mitochondrial biology in eye diseases, summarizes the current state-of-the-art in ocular MT, whilst discussing challenges and future direction of bringing MT into clinical practice.

## Highlights

• Comprehensive overview of mitochondrial function in ocular disease.
• Summary of current mitochondria-targeted therapies for ocular mitochondrial dysfunction.
• Insights into therapeutic potential of mitochondrial transplantation for ocular metabolic disorders.
• Key challenges and future directions for clinical translation in context of ocular therapeutics.

## Graphical abstract

**Figure:**
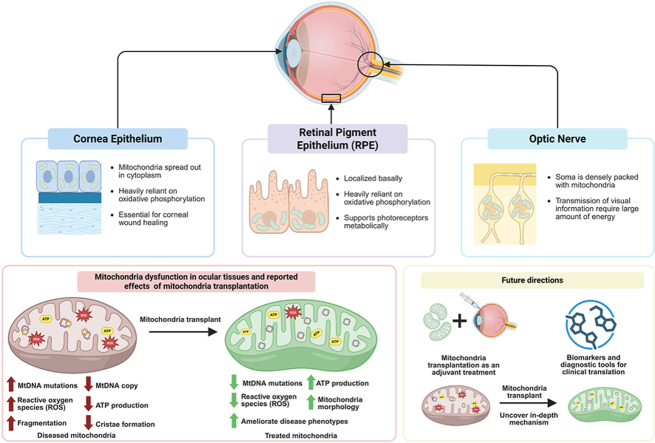

## 1 Introduction

### 1.1 Mitochondrial structure and function

Mitochondria are double-membraned tubular organelles (0.5–3 μm), essential for cellular energy production and metabolic regulation. Their structure includes an outer membrane (OM) and an inner membrane (IM), both composed of phospholipid bilayers and proteins. The OM facilitates transport of metabolites, proteins, and ions via porins such as the Voltage-Dependent Anion Channel (VDAC) [1]. Furthermore, it also plays a role in calcium signalling with the endoplasmic reticulum. It hosts the translocase of outer membrane (TOM) complex, which facilitates the import of nuclear-encoded proteins into the mitochondria. Membrane-embedded proteins involved in mitochondrial dynamics, including Fis1 and Fis2, which regulate fission, and Mitofusin, which mediates fusion, are also present in OM. Additionally, key apoptosis regulators such as Bcl-2, Bax, and Bak are localized to the OM, where they modulate cell survival and programmed cell death. The intermembrane space (IMS) between the membranes plays a key role in oxidative phosphorylation: the electron transport chain (ETC), composed of Complexes I–V, pumps hydrogen ions across the IM to generate an “ion motive force” for Adenosine-triphosphate (ATP) synthesis. Hence, disorders that primarily affect the ETC and its ATP production are referred to as mitochondrial diseases, which subsequently result in metabolic defects [2]. ETC comprises of five complexes - complex I (NADH: ubiquinone oxidoreductase), complex II (succinate dehydrogenase), complex III (CoQ-cytochrome c reductase), complex IV (cytochrome *c* oxidase), and complex V (ATP synthase). Ubiquinone (coenzyme Q10) and cytochrome *c* shuttle electrons between protein complexes and are important therapeutic targets for mitochondria-based treatments [3]. Reactive oxygen species (ROS) are predominantly produced at Complexes I and III and are detoxified by antioxidant enzymes like superoxide dismutase (SOD) within the IMS [4]. Excess ROS can lead to apoptosis, with cytochrome *c* binding cardiolipin triggering the apoptotic cascade [5,6]. Cardiolipin (CL) is a multifunctional protein that plays roles in mediating mitochondrial biogenesis, mitochondrial dynamics, and forming ETC complexes, and thus an important target for developing mitochondrial therapeutics. In contrast, the inner membrane (IM) is highly impermeable and requires special proteins for transportation of molecules and ions [7]. Cristae are produced by the infoldings in the IM which undergoes continuous remodelling in a bid to regulate its surface area to the needs of energy production and demands of the cells. Hence the number of mitochondria and cristae in each organelle is variable depending upon the energy needs of that cell type [8,9]. Mitochondrial matrix is enclosed within the IM and has the mitochondrial DNA genome and enzymes for critical functions such as oxidation of pyruvate, fatty acid and citric acid cycle. Besides being the energy hub of the cell, mitochondria also critically regulate cell signaling and death [10], biosynthetic metabolism, heme and steroid biosynthesis, apoptosis as well as signal transduction [11].

Mitochondrial DNA (mtDNA), a double-stranded circular molecule, is unique to each cell and is maternally inherited. MtDNA orchestrates various cellular functions and mitochondrial integrity. Transmission of mutated mtDNA copies and replication errors lead to development of mtDNA mutations. Additionally, lack of histones, inefficient repair system, and proximity to ETC (site of ROS production), renders mtDNA more susceptible to oxidative damage than nuclear DNA (nDNA) [12]. During energy production under normal instances, approximately 2–5 % of the oxygen is incompletely reduced, leading to elevated ROS and superoxide production.

To overcome the endogenous ROS and exogenous oxidative stress, mitochondria exhibit dynamic pleomorphism to exercise quality control which is crucial to maintain physiological state of the cell. This is performed by antioxidant enzymes, continuous process of mitochondrial biogenesis through fusion and fission of adjacent mitochondria, and mitophagy, which removes defective mitochondria for later repair. To preserve cellular homeostasis, cells have been shown to physiologically extrude damaged or senescent mitochondria, a process that aids to prevent apoptosis initiation [13]. Retinal ganglion cell axons shed unwanted mitochondria at the optic nerve head, which are internalized and degraded by adjacent astrocytes [14]. Mitochondrial transfer between retinal photoreceptors can also occur through intercellular tunneling nanotubes (TNTs), which ensures normal metabolism and function of cells under light stimulation. These dynamic processes work synergistically to maintain the mitochondrial health in diseased conditions, and their dysfunction leads to defective bioenergetics within the cell which ultimately cause diseases of mitochondrial respiratory chain.

### 1.2 Topographical and functional differences in mitochondria within eye

Mitochondria play a vital role in maintaining ocular health, with their topographical distribution and density varying across eye tissues based on metabolic demands and reliance on oxidative phosphorylation (OxPhos).

In the anterior segment, mitochondria are present in corneal stromal fibroblasts, supporting wound healing and stromal integrity. While corneal epithelial cells primarily rely on OxPhos for energy, stromal fibroblasts favour glycolysis [15]. In the lens, mitochondria are restricted to the anterior epithelium, as organelles are lost during lens fibre differentiation. Being avascular, the lens depends on aqueous humour for nutrition where it metabolize glucose mainly through anaerobic glycolysis [16].

In the retina, mitochondrial localization reflects functional diversity. Inner retinal Müller cells are glycolytic [17] and exhibit evenly distributed mitochondria [18]. Retinal ganglion cells (RGCs), which depend heavily on OxPhos, have mitochondria densely packed in their soma and unmyelinated axons. The myelinated optic nerve contains fewer mitochondria due to lower energy demands [19]. This metabolic reliance makes RGCs more vulnerable to mitochondrial damage than glycolytic retinal cells.

Photoreceptors (PRs) in the outer retina contain mitochondria concentrated in the inner segments and synaptic terminals, supporting their high metabolic rate required for phototransduction and outer segment renewal. PRs favour glycolysis for rapid ATP turnover and possess mitochondria in the ellipsoid region that function as microlenses to enhance light focusing [20]. In contrast, the retinal pigment epithelium [21], a monolayer of postmitotic cells between PRs and the choroid, relies heavily on OxPhos. RPE mitochondria are densely packed at the basal side, near the choriocapillaris, facilitating efficient oxygen utilization. RPE cells support the neural retina metabolically, forming a symbiotic ecosystem where PRs consume glucose and produce lactate, which RPE cells use as fuel [22]. RPE mitochondria generate ATP via the TCA cycle, β-oxidation and OxPhos, but their high metabolic activity and basal ROS levels make them susceptible to mitochondrial damage. Daily phagocytosis of PR outer segments further stresses RPE mitochondria, increasing mtDNA damage. Mitochondrial number in RPE cells is dynamic, ranging from 734 ± 170 (23.2 %), adapting to energy demands through biogenesis and mitophagy [23]. These processes are regulated by Peroxisome proliferator-activated receptor gamma coactivator-1 alpha (PGC-1α), which co-activates transcription factors such as Nuclear Respiratory Factor 1 (NRF-1), Peroxisome Proliferator Activated Receptor Alpha (PPARα), and Transcription Factor A, Mitochondrial (TFAM) to drive mitochondrial gene expression [24].

Altogether, mitochondria across ocular tissues operate in a coordinated manner to sustain visual function. Their dysfunction underlies several debilitating eye diseases, as explored in the following section.

### 1.3 Mitochondrial dysfunction within eye

Although individual mitochondrial diseases affecting the eye are relatively rare, they are collectively more prevalent, with an estimated adult prevalence of approximately 1 in 4300. Notably, over half of affected individuals exhibit ocular manifestations, which may include optic neuropathies, retinal degeneration, ptosis, and ophthalmoplegia [25]. These features are observed across a wide spectrum of conditions, ranging from rare syndromes such as Leber's Hereditary Optic Neuropathy (1 in 30,000 to 50,000), Kearns-Sayre Syndrome (1 in 4000), and Progressive External Ophthalmoplegia, to more common diseases associated with mitochondrial dysfunction, including glaucoma, diabetic retinopathy, and age-related macular degeneration [26,27].

Mitochondrial diseases predominantly affect organs with high metabolic demands [28], making ocular tissues such as the retina, optic nerve, and cornea particularly vulnerable due to their reliance on OxPhos. Mitochondrial dysfunction in the eye manifests as either primary or secondary mitochondrial diseases, both contributing to significant visual impairment, Pathophysiologically, **primary mitochondrial diseases (PMD)** arise from germline mutations in any of the 37 mitochondrial DNA (mtDNA) genes or in over 300 nuclear DNA (nDNA) genes encoding components of the electron transport chain (ETC), hence disrupting OxPhos and cellular bioenergetics [29]. In contrast, **secondary mitochondrial dysfunction (SMD)** results from non-genetic factors—such as aging, inflammation, or exposure to mitotoxic drugs—that elevate oxidative stress and induce mtDNA damage. SMD may also accompany hereditary non-mitochondrial disorders involving non-ETC mitochondrial functions like fatty acid oxidation [30] and the Krebs cycle [31].

Central to most ocular SMDs is the accumulation of ROS-induced mtDNA mutations [32]. Mitochondrial genome instability is a hallmark of these diseases, with mtDNA mutations occurring 10–20 times more frequently than nDNA mutations due to mtDNA's proximity to ROS and its inefficient, error-prone repair mechanisms [33]. The role of ROS production within the mitochondria could be a double-edged sword; while moderate ROS levels are essential for energy production and redox signalling, excessive ROS leads to oxidative stress, overwhelming cellular detoxification and triggering degeneration or cell death. Ocular tissues are uniquely exposed to both endogenous oxidative stress from high OxPhos activity and exogenous stressors such as light exposure, smoking, and diet. This cumulative burden contributes to the pathogenesis of several eye diseases, including age-related macular degeneration (AMD), diabetic retinopathy (DR), glaucoma, optic neuropathies, and corneal disorders.

#### 1.3.1 Corneal disorders

Corneal epithelial and endothelial cells are densely populated with mitochondria to support the high metabolic demands of the ocular surface. Akin to all SMDs, mitochondrial dysfunction in the cornea is influenced by environmental factors such as aging, diet, blue light exposure, diabetes, and autoimmune conditions like Sjögren's syndrome [34]. Among these, ultraviolet radiation, particularly UVA, is a major contributor inducing mtDNA damage via photodynamic mechanisms [35]. The corneal stroma harbours the highest levels of the common mtDNA deletion mutation (mtDNACD4977), predominantly attributed to UV exposure. Dry eye disease, often resulting from corneal epithelial cell (CEC) damage, is driven by ocular surface inflammation and ROS-induced oxidative stress [36,37]. Mitochondrial dysfunction in this context impairs tear production and has been histopathologically linked to lacrimal gland dysfunction [38]. Corneal dystrophies, notably Fuchs endothelial corneal dystrophy (FECD) also exhibit mtDNA damage [35]. *In vitro* studies report diseased CECs have reduced mitochondria bioenergetics, fragmentation and loss of mitochondria membrane potential [15,39]. This leads to activation of mitochondria caspase-mediated apoptosis and loss of CECs, which is a key event in FECD. Consequently, fluid balance and functional homeostasis of the cornea are lost, promoting corneal edema and visual disability.

#### 1.3.2 Optic nerve disorders

##### 1.3.2.1 Glaucoma

Retinal ganglion cells (RGCs) are composed of long axons that contain a high concentration of mitochondria required for generating energy. This makes them one of the most metabolically active tissues in the body and therefore, they are particularly susceptible to mitochondrial diseases. Experimental evidence suggests that mechanical stress, chronic hypoperfusion and hypoxia induced mitochondrial oxidative stress secondary to elevated intraocular pressure causes degeneration in RGCs and retina by adversely affecting their bioenergetics [40,41]. Light-induced ROS has been shown to cause mtDNA abnormalities and mitochondrial dysfunction in animal models of glaucoma [42]. Additionally, trabecular meshwork cells play a key role in regulating the outflow of aqueous humour from the anterior chamber and maintaining normal intraocular pressure. These cells are highly sensitive to damage caused by ROS targeting mtDNA. In patients with glaucoma, they also show a significant reduction in antioxidant capacity, which further impairs their function [43]. Thus, secondary mitochondrial dysfunction is the underlying pathology in glaucoma.

##### 1.3.2.2 Hereditary optic neuropathies

Hereditary Optic Neuropathies are the only ocular primary mitochondrial diseases directly affecting mtDNA or nDNA genes resulting in ETC functional defects [44]. Autosomal dominant optic atrophy (ADOA) and Leber's hereditary optic neuropathy (LHON) are the most common types of mitochondrial optic neuropathies [45]. The primary aetiology encompasses demyelinating, inflammatory, ischaemic, traumatic, compressive, toxic/nutritional and hereditary causes [46]. This leads to mtDNA or nDNA mutations in RGCs resulting in dysfunction and degeneration, with symptoms comprising reduced visual acuity, colour vision deficits, scotomas and pale optic disc with attenuation of retinal nerve fibre layer. While 50–75 % of ADOA is caused by mutations in the nuclear gene OPA1 [47], which plays a critical role in mitochondrial fusion; LHON is caused by mutations in both mtDNA (top three most common mtDNA mutations being m.3460G > A, m.11778G > A and m.14484T > C). Nuclear gene (DNAJC30) mutation causes recessive LHON [48]. Variable or incomplete penetrance is known to occur and is influenced heavily by environmental factors [49].

Chronic Progressive External Ophthalmoplegia (CPEO) is a complex PMD which may present as part of a generalized mitochondrial myopathy. While ophthalmologic manifestations involve extraocular muscles and have clinical features of strabismus, ptosis, pigmentary retinopathy and progressive external ophthalmoplegia; multi-system involvement is common in 50 % of patients and include gastrointestinal dysfunction, migraines, and cardiac conduction defects [50]. Autosomal inheritance of six nuclear genes has been implicated, including TYMP, ANT1, PEO1, POLG, POLG2, and even OPA1 [51].

#### 1.3.3 Retinal disorders

##### 1.3.3.1 Age related macular degeneration (AMD)

AMD is classified as a secondary mitochondrial disease (SMD), with mitochondrial dysfunction in RPE cells playing a central role in its pathogenesis. This dysfunction arises secondary to aging, genetic predisposition, environmental factors, and lifestyle influences [52]. AMD affects the macula, the region responsible for central vision and rich in photoreceptors. RPE cells in the macula are chronically exposed to elevated oxidative stress, with ROS levels significantly higher than in peripheral retina [53]. This leads to cumulative mtDNA damage, heteroplasmic mutations, and progressive mitochondrial dysfunction [54]. Drusen, a hallmark of AMD, contains lipids and reactive species can exacerbate mtDNA damage. Experimental evidence from primary RPE cultures and donor eyes with early AMD reveals diminished mitochondrial respiration, reduced ATP production, and structural abnormalities including decreased mitochondrial number, size, and disrupted cristae [55]. Defective mitochondrial biogenesis, fusion, and impaired autophagy/mitophagy have also been reported, along with reduced expression of ETC protein subunits [56].

AMD disrupts the metabolic symbiosis between RPE and photoreceptors. Under normal conditions, RPE cells metabolize lactate from photoreceptors, suppressing their own glycolysis and facilitating glucose transport to PRs. In AMD, mitochondrial damage forces RPE cells to rely on glycolysis, reducing glucose availability for PRs. This leads to decreased lactate production, further impairing RPE metabolism and creating a vicious cycle of energy deprivation and photoreceptor degeneration [57].

Several mitochondrial DNA variants have been implicated in AM, particularly within genes encoding components of the ETC. Notable pathogenic changes include MT-ND2 (m.4917A > G), MT-ND4 (m.11812A > G and m.11778G > A), MT-ND6 (m.14233A > G), and MT-ATP6 (m.8993T > G) [58]. These mutations may disrupt oxidative phosphorylation, contributing to retinal bioenergetic deficits and increased susceptibility to oxidative stress. Beyond single-nucleotide changes, specific mtDNA haplogroups have shown differential associations with AMD phenotypes. Haplogroups J and U have been linked to soft drusen formation and RPE abnormalities, suggesting a predisposition to early structural changes [59]. In contrast, haplogroup H appears to confer a protective effect against AMD progression [60]. Additionally, pathogenic variants within the non-coding D-loop region of mtDNA, critical for replication and transcriptional regulation, have demonstrated strong associations with late-stage AMD. These variants may influence mitochondrial copy number and susceptibility to oxidative damage, thereby exacerbating retinal degeneration. Collectively these findings underscore mitochondrial dysfunction in RPE as a key driver of AMD pathogenesis.

##### 1.3.3.2 Diabetic retinopathy (DR)

Mitochondrial dysfunction in DR is driven by a complex interplay of metabolic, oxidative, inflammatory, and epigenetic factors triggered by chronic hyperglycaemia [61]. In the diabetic retina, excessive glucose flux overwhelms glycolytic and mitochondrial pathways, leading to increased electron leakage from the ETC and the generation of ROS, particularly superoxide at complex III [62]. This oxidative burden damages mtDNA, lipids, and proteins, and impairs ETC activity, culminating in bioenergetic failure and apoptotic signalling [63].

Hyperglycaemia also activates NADPH oxidase isoforms, notably Nox2 and its regulatory subunit Rac1, which amplify cytosolic ROS and exacerbate mitochondrial injury [64]. Concurrently, antioxidant defences such as glutathione are depleted, reducing the retina's capacity to neutralize ROS and maintain redox homeostasis. These events disrupt mitochondrial membrane potential, reduce mitochondrial number and volume, and impair cristae integrity across multiple retinal cell types, including microvascular endothelial cells, RPE, Müller glia, and ganglion cells studies [65]. Inflammatory signalling further compounds mitochondrial dysfunction. Damaged mitochondria released into the vitreous during mitophagy can trigger immune responses. They activate Toll-like receptors on macrophages and RPE cells, leading to the production of inflammatory signals like IL-1β and TNF-α. Mitochondrial function also regulates macrophage polarization, and recent transcriptomic analyses have identified mitochondria-related genes (MRGs) such as PTAR1 and SLC25A34 as biomarkers linked to immune infiltration and insulin signalling pathways in DR [66].

Importantly, mitochondrial dysfunction in DR persists despite glycaemic normalization, reflecting a phenomenon of “metabolic memory” [67]. Epigenetic modifications, including DNA methylation, histone acetylation, and accumulation of advanced glycation end-products (AGEs) sustain transcriptional repression of mitochondrial biogenesis and antioxidant genes [68]. For example, acetylation of Mitofusin 2 (Mfn2), a key mitochondrial fusion protein, impairs its GTPase activity and mitophagy flux and leads to mitochondrial fragmentation and accumulation of damaged organelles [65].

Together, these mechanisms underscore the multifactorial nature of mitochondrial impairment in DR and highlight mitochondria as both targets and amplifiers of retinal pathology. Therapeutic strategies aimed at restoring mitochondrial integrity, enhancing mitophagy, and modulating inflammatory signalling may offer promising avenues for halting or reversing disease progression.

### 1.4 Overview of current mitochondria targeted treatment for eye diseases

**Fig. 1:**
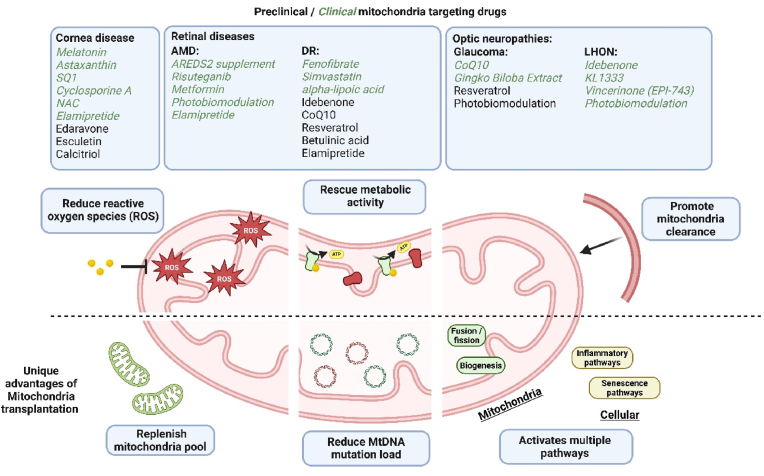
**Overview of mitochondrial therapies targeting metabolic dysfunction in ocular diseases.** Mitochondrial drugs used in various ocular diseases classified by their underlying mechanism of action in mitochondrial dysfunction including reduction of reactive oxygen species (ROS), restoration of metabolic activity, promotion of mitophagy, and replenishment of the mitochondrial pool. Drugs currently used in clinical trials are in green italic, while all other drugs are explored in research settings. Mitochondrial transplantation (MT) is highlighted for its unique ability to replenish functional mitochondria, reduce mtDNA mutation load, and activate multiple redox-sensitive pathways. Key mitochondrial processes such as fusion/fission, biogenesis, and senescence are illustrated to contextualize therapeutic targets.

Therapeutic strategies aimed at restoring mitochondrial function in ocular diseases such as age-related macular degeneration (AMD), diabetic retinopathy (DR), hereditary optic neuropathies, glaucoma, and dry eye syndrome are actively under investigation to improve visual outcomes. Despite growing interest, there are currently no FDA-approved treatments specifically targeting mitochondrial dysfunction in the eye. Emerging therapies focus on modulating key mitochondrial pathways, including stabilization of membrane potential, regulation of redox signalling, attenuation of oxidative stress, inhibition of apoptosis, and enhancement of mitochondrial biogenesis. These approaches are discussed in this section (Fig. 1).

#### 1.4.1 Corneal disorders

Antioxidants remain the cornerstone of mitochondrial-targeted therapies for **dry eye disease**. These agents primarily act by activating nuclear factor erythroid 2–related factor 2 (Nrf2), which is a master regulator of cellular antioxidant defense. Nrf2-targeting compounds such as Edaravone [69], Esculetin [70], and Calcitriol [71] have demonstrated promising *in vitro* results by upregulating antioxidant enzymes such as heme oxygenase-1 (HO-1) and glutathione peroxidase 1 (GPX1). This enhances cellular resilience against oxidative stress, mitigates hyperosmolarity-induced damage, and inhibits apoptosis by suppressing cytochrome *c* release from mitochondria. SkQ1 (Visomitin, Mitotech SKQ, Luxembourg), a mitochondria-targeted short-chain quinone, accumulates within mitochondria and promotes antioxidant expression [72]. Clinical trials have shown its efficacy in improving tear film stability and alleviating dry eye symptoms. Cyclosporine A (CsA), a well-established immunomodulatory agent, enhances mitochondrial function by binding cyclophilin D (CypD). Thereby stabilizing mitochondrial membrane permeability and modulating mitochondrial dynamics. Clinical studies have demonstrated CsA's ability to improve cellular survival and integrity in dry eye patients [73,74].

Beyond dry eye disease, mitochondrial-targeted therapies are gaining attention in the treatment of **Fuchs endothelial corneal dystrophy (FECD)** which is one of the most common causes of corneal blindness worldwide. It is characterized by the progressive loss of corneal endothelial cells, which are essential for maintaining the cornea's fluid balance and optical clarity. As these cells degenerate, the cornea becomes edematous and loses its transparency, leading to visual impairment. Traditionally, surgical interventions such as endothelial keratoplasty have been the mainstay of treatment. However, limitations including donor tissue scarcity and postoperative complications have prompted the search for non-surgical alternatives [75]. In this context, therapies that target mitochondrial dysfunction are being explored as a new way to protect corneal function. By reducing endothelial cell death and restoring cellular homeostasis, these approaches may help slow disease progression. Elamipretide, a mitochondrial membrane stabilizer, enhances mitochondrial integrity and function, thereby supporting cellular resilience under stress [76]. N-acetyl cysteine (NAC), a potent antioxidant and free radical scavenger, mitigates oxidative damage and preserves endothelial cell viability [77]. Sirolimus, an mTOR inhibitor, acts by suppressing apoptosis in corneal endothelial cells, offering a potential strategy to delay or prevent disease progression [78].

**Corneal wound healing**, particularly in epithelial defects caused by trauma, chemical burns, or diabetes, is highly dependent on mitochondrial integrity. Recalcitrant epithelial defects may result in delayed re-epithelialization, scarring, and corneal opacification, often necessitating surgical intervention. Coenzyme Q10 (CoQ10), an essential electron carrier in the respiratory chain, has been shown to enhance mitochondrial function and promote corneal healing following UVB exposure in both *in vitro* and *in vivo* models [79]. Similarly, SkQ1 has demonstrated efficacy in accelerating corneal epithelial repair under UV and mechanical injury by supporting cell survival and reviving metabolic balance [69]. Together, these agents represent a shift toward non-surgical interventions aimed at restoring corneal homeostasis.

#### 1.4.2 Optic nerve disorders

##### 1.4.2.1 Glaucoma

The hallmark of glaucoma is the vulnerability of RGCs to oxidative insults, which disrupt mitochondrial function and accelerate neurodegeneration. Antioxidant supplementation has shown promise in preclinical models, demonstrating improved oxidative balance, enhanced RGC survival, and restored axonal transport. Agents such as coenzyme Q10 (CoQ10) [80], resveratrol [81], and Ginkgo biloba extract [82] have exhibited mitochondrial protective effects in animal studies. Small-scale clinical investigations in glaucoma patients have reported improvements in visual fields and reductions in oxidative DNA damage. Despite these encouraging findings, these therapies are currently not FDA-approved or clinically validated. This limitation arises from the inability to consistently localize drugs to mitochondria at therapeutically relevant concentrations.

Intermittent hypoxia has emerged as a potential strategy to mitigate mitochondrial oxidative stress by preconditioning cells through adaptive responses. This approach upregulates antioxidant pathways and hypoxia-inducible factor offering neuroprotection to RGCs in animal models [83]. However, translation to human studies remains limited by the absence of standardized protocols.

Similarly, PBM has demonstrated neuroprotective effects in preclinical models by enhancing mitochondrial bioenergetics and preventing RGC pruning and degeneration [84]. However, clinical translation remains limited due to protocol variability, insufficient regulatory frameworks, and technical challenges in achieving consistent light delivery to the target retinal area. Inter-patient differences in ocular anatomy and light penetration further complicate standardization, underscoring the need for harmonized treatment parameters and robust clinical validation.

Consequently, no standardized mitochondrial-targeted interventions currently exist to regenerate dysfunctional RGCs in glaucoma. This underscores the need for alternative mitochondrial therapeutics, especially given the limitations of current intraocular pressure lowering treatments. While topical medications, laser therapy, and surgery can slow disease progression, they do not reverse vision loss.

##### 1.4.2.2 Optic neuropathies

Currently, the only approved treatment for Leber's Hereditary Optic Neuropathy (LHON) in the European Union is Idebenone (Raxone®, Santhera Pharmaceuticals), a quinone analogue that bypasses mitochondrial complex I dysfunction. This restores ATP synthesis and reduces apoptotic signaling. Clinical trials such as RHODOS (NCT00747487) and LEROS (NCT02774005) have demonstrated modest efficacy, with limited improvements in visual acuity among treated patients [85].

Other quinone analogues with enhanced potency *in vitro* are under early-phase investigation. These include KL1333, an NQO1-dependent NAD^+^ modulator, and EPI-743 (vatiquinone; Vincerinone, Edison Pharmaceuticals), both currently in phase I trials for mitochondrial disorders [86].

PBM has also been explored as a non-invasive therapeutic approach. By targeting cytochrome *c* oxidase with near-infrared light, PBM aims to enhance mitochondrial bioenergetics and upregulate antioxidant defenses. While *in vitro* studies have shown promise, clinical trials have failed to demonstrate significant benefit, largely due to challenges in patient fixation and consistent retinal targeting.

Importantly, none of these therapies are curative, as they do not correct the underlying mitochondrial DNA mutations. In this context, transplantation of healthy mitochondria represents a novel and potentially transformative strategy. By reducing mutational load and restoring cellular bioenergetics, mitochondrial transplantation may alleviate ocular symptoms and improve visual outcomes in LHON patients. This approach warrants further investigation as a next-generation therapy for inherited mitochondrial optic neuropathies.

#### 1.4.3 Retinal disorders

##### 1.4.3.1 Age-related macular degeneration (AMD)

Mitochondrial-targeted treatments for dry AMD remain limited, with current strategies largely confined to nutritional supplements and antioxidants, as evidenced by the Age-Related Eye Disease Studies (AREDS 1–2) [87]. These interventions aim to alleviate oxidative stress in RPE mitochondria by preventing lipid peroxidation and upregulating antioxidant gene expression. Their primary goal is to slow the progression from intermediate AMD to advanced stages, such as geographic atrophy (GA) or choroidal neovascularization. The 5-year AREDS data demonstrated a 25 % reduction in the risk of advanced AMD among high-risk participants, with benefits sustained up to 30 % at 10-year follow-up [88,89]. Central visual loss was reduced by 19 %, likely due to slowed GA progression toward the fovea, augmenting the natural phenomenon of foveal sparing [90]. However, AREDS supplementation does not prevent the onset of early AMD nor its progression to the intermediate stage.

For GA, an advanced form of dry AMD, investigational therapies targeting mitochondrial membrane stabilization and biogenesis are under clinical evaluation. Elamipretide (MTP-131), a mitochondria-targeted peptide, binds cardiolipin to preserve mitochondrial homeostasis and protect against ROS-induced damage. In the phase I ReCLAIM trial, daily subcutaneous injections of 40 mg MTP-131 in patients with non-exudative AMD and non-central GA (NCGA) led to improvements in low-luminance visual acuity (LLVA) and a mean reduction in NCGA area of ∼0.13 mm^2^. In quiescent exudative AMD, 54 % of eyes gained ≥6 BCVA letters and 31 % gained ≥10 letters. However, the subsequent phase II trial failed to meet its primary endpoint, likely due to significant baseline photoreceptor loss and a high incidence (86 %) of adverse effects [91].

Risuteganib (Luminate), an integrin modulator, supports mitochondrial function in RPE cells by mitigating oxidative stress and preserving energy production. In a phase II randomized trial involving patients with intermediate AMD (n = 42), intravitreal Risuteganib (1 mg) resulted in an 8-letter BCVA gain in 48 % of treated subjects, compared to 7.1 % in the sham group (p = 0.013) [92]. Despite these promising results, no phase III trials are currently underway.

Metformin, a widely used oral hypoglycemic agent, activates AMP-activated protein kinase (AMPK), which in turn stimulates PGC-1α, the master regulator of mitochondrial biogenesis. Retrospective case-control data suggest a protective effect against AMD progression [93]. However, the METforMIN phase II trial (NCT02684578) did not demonstrate efficacy in slowing GA progression. Limitations included a high proportion of participants with large baseline GA, systemic adverse effects leading to early withdrawals, and suboptimal dosing strategies that compromised statistical power.

Use of infrared and near-infrared spectrum radiation in therapeutic range known as photo biomodulation (PBM), has shown to act via cytochrome *c* oxidase to enhance mitochondrial electron transport and ATP production. A systematic review and meta-analysis of randomized clinical trials found that PBM led to statistically significant but clinically modest improvements in BCVA (mean 1.76 letters) and drusen volume, with no effect on geographic atrophy. All included studies had high risk of bias and insufficient sample sizes for reliable conclusions [94]. While PBM showed transient anatomical and functional gains in small sample sized clinical studies, these required repeated treatments and did not translate into sustained clinical benefit^,^ [95]. PBM shows limited efficacy in AMD due to the disease's multifactorial nature. This therapy doesn't address key drivers like complement dysregulation, lipid accumulation, or chronic inflammation, and its effects are transient without structural or metabolic remodelling. Additionally, poor retinal targeting, heterogeneous cellular responses, and underpowered clinical trials further dilute its therapeutic impact.

Overall, current mitochondrial-targeted therapies for AMD primarily slow disease progression but fail to rejuvenate degenerated RPE cells. Repeated administration often leads to injection-site reactions and systemic adverse effects. Mitochondrial dysfunction in diseased RPE cells persists and may worsen over time, contributing to continued degeneration. This underscores a critical unmet need for more effective, durable therapies capable of restoring mitochondrial integrity and cellular function in AMD.

##### 1.4.3.2 Diabetic retinopathy (DR)

Mitigation of oxidative stress, mitochondrial dysfunction, and endothelial injury within retinal cells represents a compelling therapeutic axis in DR. A range of antioxidants including Idebenone [96], coenzyme Q10 (CoQ10) [97], alpha-lipoic acid [98], resveratrol [81], and betulinic acid [99] are under investigation as promising therapeutic avenues. They have demonstrated ability to neutralize ROS *in vitro* and attenuate visual impairment in preclinical DR models, though their clinical translation remains limited.

Pharmacologic agents such as Fenofibrate [100] and Simvastatin [101] have shown promise in promoting mitochondrial biogenesis by suppressing ROS generation under hyperglycemic conditions. Acting through peroxisome PPAR pathways and their coactivators, these drugs have been reported to prevent retinal endothelial cell apoptosis and pericyte dropout in experimental models [101]. Despite encouraging findings, clinical trials evaluating these interventions have been constrained by small sample sizes and short follow-up durations (typically 6–12 months), which are insufficient to capture the long-term trajectory of a progressive disease like DR.

Elamipretide is currently being evaluated as a topical ophthalmic formulation for diabetic macular edema. While early-phase trials are ongoing, no mitochondria-directed therapy has yet demonstrated definitive efficacy in halting DR progression [76].

The concept of ‘metabolic memory, whereby persistent mitochondrial dysregulation drives retinopathy despite optimal glycemic control, underscores the need for early intervention. Protecting mitochondrial integrity during periods of normoglycemia may help restore biogenic signaling and prevent further retinal damage. In this context, regulation of mitochondrial homeostasis at early disease stages could offer a strategy to arrest or delay retinopathy progression.

While MT has not yet been investigated in the context of DR, its potential to restore bioenergetic function and rescue retinal cells from hyperglycaemia-induced mitochondrial damage warrants exploration. Given the central role of mitochondrial dysfunction in DR pathogenesis, MT may offer a promising therapeutic avenue for future investigation.

### 1.5 Mitochondrial transplant for eye diseases

This innovative therapeutic modality involves the introduction of either autologous or heterologous functional extracellular mitochondria *in vitro* into cells or *in vivo* in tissues to replace the diseased or defective mitochondria caused by genetic mutations, oxidative damage, or advanced age [102].

Mitochondrial transplant as a treatment for ocular diseases is an emerging yet exciting field. Extracellular mitochondria (isolated mitochondria) transfer in eye diseases encompasses either contact-dependent mitochondria transfer via co-incubation with mesenchymal stem cells or contact-independent transfer of isolated mitochondria to enable metabolic recovery of diseased cells in various ocular diseases [103]. Overview of the mitochondria transplant field in the eye is presented in Table 1.

**Table 1:** Compiled contact independent mitochondria transfer studies in eye field, listed chronologically.

Year	Disease	Mitochondria source	Isolation method	*In vitro*/*In vivo* model	Mode of delivery (quantity of mitochondria)	Mitochondria transplant effects
2020 [104]	Optic Nerve Injury	Rat Liver	Tissue minced, homogenized with 30 mL Elvehjem glass potter and isolated with published protocol [105]	Optic nerve crush in 30–60 days old *Lister Hooded* Rat model	Intravitreal injection (5 μl of 0.25 mg/mL isolated mitochondria in saline)	• Enhanced a-wave and b-wave amplitude 1 day post transplantation • Significant increase in number of Tuj1+ cells 14 days after treatment (not observed at 28 days timepoint) • Axon length increased overall 28 days post treatment • Whole retina showed increased spare capacity 3 days post treatment
2022 [106]	Retinitis Pigmentosa	Rat Liver from eight- to twelve-week-old RCS rats	Homogenization by handheld homogenizer followed by discontinuous sucrose density gradient centrifugation	Four weeks old RCS rats	Intravitreal injection (50 μg or 100 μg mitochondria in 2 μL DMEM)	• 5 weeks post-transplantation of 50ug and 100ug mitochondria, ONL cell number increased by 118 % & 173 % respectively. • Retina thinning effects were ameliorated • Improved Visual Evoked Potential N1 latency 2 weeks but not 5 weeks post transplantation
2022 [107]	Ocular ischemia	C57BL/6 or BALB/c mouse livers	Homogenization followed by differential centrifugation + 10 μM membrane filtration	661W photoreceptor cells treated with H2O2	Co-incubation for 24h (0.5–2 million mitochondria)	• Oxidative stress increases isolated mitochondria uptake • Oxidatively stressed cells promote transplanted mitochondria survival
2023 [108]	AMD	Umbilical cord mesenchymal stem cells	Cells homogenized using disposable 1 ml syringe, followed by Mitochondria/Cytosol Fraction kit (Abcam)	TBHP/H2O2 senescence-induced ARPE-19 cells	Co-incubation for 5 days (10 μg mitochondria per 1 × 105 cells)	• Inflammatory modulatory: Reduced TNF-α, IL-8 mRNA levels, (2) reduced NF-κB phosphorylation • Reduced expression of senescence proteins (p16, p21) • Altered mitochondria dynamics proteins (PINK1, PARKIN, DRP1, MFN1) • Reduced cell size in senescence-induced cells
2023 [109]	Fuchs endothelial corneal dystrophy (FECD)	HEK293T cells	Homogenized then filtered through 40 μm, 10 μm and 5 μm filters	FECD explants from patients with late stage FECD	Co-incubation for 2h, 3h or 48h (0.2 mg/mL mitochondria in DMEM)	• 1.5-2-fold increase in mitochondria membrane potential • 3-fold decrease in oxidative stress per cell after 48h • 3-fold decrease in mitophagy activity per cell after 2h • ∼4-fold decrease in percentage of apoptotic cells
2024 [110]	Corneal epithelial defects	ARPE-19 cells or Mouse liver from C57BL/6 mice	Homogenization followed by differential centrifugation	Corneal epithelial cells isolated from humans (hCECs)/NaOH induced cornea burn in C57BL/6 mice	*In-vitro*: Co-incubation for 24h (not specified) *In-vivo*: Isolated mitochondria diluted in 25ul buffer, provided as eyedrop every 30 min for 3 h over 4 days (not specified)	• Promotes proliferation: 40 % increase in wound closure (2) 12 % increase in Ki67+ cells • Closure of alkali burn in 4 days (50 % more closure than control) and reduced cornea thickness
2024 [111]	AMD	Bone Marrow Mononuclear Cells	Cells homogenized using disposable 1 ml syringe, followed by Mitochondria/Cytosol Fraction kit (Abcam)	Oligomeric amyloid-beta (oAβ) in ARPE-19 and six-week-old C57BL/6J mice	*In-vitro*: Co-incubation for 63h (10 μg mitochondria per 1 × 105 cells) *In-vivo*: Intravitreal injection (not specified)	• Decreased mitochondria mass • Promoted mitochondria membrane potential • Increased ATP levels • Decreased cellular and mitochondria ROS • Attenuated ZO-1 protein loss in-vitro and in-vivo • Akt signalling activated post-transplant in-vitro
2024 [112]	LHON	Hela cells or BALB/c mice heart tissue with *PARKIN* mRNA-loaded nanoparticle (mNP-mito)	Mitochondrial Isolation Kit (not specified)	Rotenone-treated cell & animal model	*In-vitro & in-vivo*: Co-incubation for 24h (10μg)	• Promote mitophagy of damaged mitochondria • *In-vitro:* Recovered mitochondria membrane potential, mitochondria ROS levels, complex I content (up to 3-fold) and ATP levels (2-fold) • *In vivo*: improved ATP levels, retina thickness and vision • Transplanted mNP-mito did not trigger systemic and local inflammation after 7 days

#### 1.5.1 Contact-dependent MT

This process involves the transmission of mitochondria through direct contact between donor and recipient cells, typically via specialized structures such as tunnelling nanotubes (TNTs), which are dendritic outpouchings. Mitochondrial transfer may also occur through direct adhesion mechanisms, including gap junctions or endocytosis.

There is growing *in vitro* and *in vivo* evidence supporting the role of tunneling nanotubes (TNTs) in facilitating mitochondrial transfer between ocular cell types. Studies using ARPE19, an immortalized retinal pigment epithelium cell line, have shown that these cells can exchange mitochondria with one another through TNTs. Moreover, ARPE19 cells are capable of receiving mitochondria from donor cells such as mesenchymal stem cells, highlighting the potential of TNT-mediated transfer as a mechanism for mitochondrial rescue and intercellular support in retinal disease models. In fact, the expansion of literature's understanding of MT using TNTs in the eye has been largely precipitated using Mesenchymal stem cells (MSCs) as a donor source. Studies utilizing a co-culture model of rotenone-induced mitochondrial dysfunction were also able to effectively show the transportation of healthy mitochondria from MSCs to human corneal epithelial cells (CECs) [113]. Mitochondrial parameters such as basal respiration, ATP production and maximal respiration were significantly elevated after co-culturing rotenone treated CECs with MSCs. Additionally, MT from human MSCs to rabbit cornea was also shown to greatly promote wound healing [110]. Further, the benefits of contact driven MT were also evident in bioenergetically compromised photoreceptor cells. *In vivo* and *in vitro* data reports donor mitochondria promoted mitochondrial function, enhanced proliferative capacity, and modified cellular metabolic pathways. It is worth noting that donor mitochondria were found to remain viable for up to 8 days after transplant, following which they were either degraded by lysosomes or excreted via extracellular vesicles. Contact-dependent MT has also been demonstrated between murine bone marrow MSCs and Müller cells. Donor MSCs transplanted in the subretinal space resulted in MT in outer nuclear layer in retina. Gliosis suppression along with reduced apoptosis and improved retina function were noted up to 6 weeks post transplantation [114]. The underlying rescue mechanism is hypothesized to be augmentation of mitochondria fusion, and mtDNA enhancement. Release of brain-derived neurotrophic factors, known to protect against retinal degeneration, was also upregulated.

#### 1.5.2 Contact-independent MT

This process refers to the delivery of mitochondria to recipient cells without direct physical contact with the donor cell. In this process, isolated mitochondria from cells are introduced into the extracellular environment—such as interstitial fluid, plasma, cerebrospinal fluid, saliva, or cell culture media—where they can be taken up by target cells.

This method of MT has been more commonly employed in recent years since therapeutic potential of artificial transplantation of metabolically active isolated mitochondria has been demonstrated in several *in vitro* experiments of cardiac [115], hepatic [116] and central nervous system diseases [117]. We discuss further issues pertaining to contact-independent transfer of isolated mitochondria in eye in the following sections:

1 Methods of mitochondrial isolation
2. Optimal source of mitochondria for ocular transplantation
3. Delivery of mitochondria into the eye
4. Efficacy of Mitochondrial Transplantation in eye disorders
5. Potential biomarkers and diagnostic tools (to assess efficacy in humans)

##### 1.5.2.1 Methods of mitochondrial isolation

Two primary methods are used for mitochondrial isolation, with **differential centrifugation** being the most common. This technique involves homogenizing host cells or tissues, followed by low-speed centrifugation to remove debris and high-speed centrifugation to pellet the mitochondrial fraction. Subsequent washing steps help purify the mitochondria. However, this method often results in contamination with other cellular components such as the endoplasmic reticulum, lysosomes, and cytoplasmic proteins. The efficiency and purity of isolation can vary depending on tissue type, as mitochondrial density differs across tissues. Therefore, the homogenization protocol must be tailored to optimize mitochondrial yield and integrity [118]. In addition to differential centrifugation, **density gradient centrifugation** is widely employed to isolate mitochondria based on their buoyant densities. This method utilizes gradients composed of isotonic sucrose or its polymers to achieve separation from other cellular fractions, yielding a purer mitochondrial preparation [119].

Alternative approaches have emerged to enhance specificity and purity. One such method is **affinity-based mitochondrial purification**, which has been successfully applied in *C. elegans*. In this technique, mitochondria are genetically modified to express a hemagglutinin (HA) tag, allowing selective isolation of mitochondrial subpopulations from limited cell numbers [120]. While effective, this strategy is less cost-efficient and has not seen widespread adoption in mitochondrial research.

A more recent innovation involves **magnetic bead-based immunopurification**, which addresses several limitations of traditional methods. This technique employs mitochondria-targeted magnetic nanoparticles conjugated with TOM20 antibodies to selectively bind mitochondria based on membrane potential. The result is a highly pure mitochondrial fraction with minimal organelle contamination [121]. However, the method is constrained by its prolonged processing time (6–8 h), the need for highly specific magnetic beads, and elevated costs, which may limit its scalability.

**Flow cytometry** has emerged as a powerful technique for mitochondrial isolation, enabling the recovery of highly pure mitochondrial fractions from crude pellets. This method allows for the discrimination and sorting of mitochondrial subpopulations based on size and membrane potential, using fluorescent probes and antibodies targeting outer mitochondrial membrane proteins. Such precision facilitates subpopulation-level analysis, which is particularly valuable for functional and translational studies. However, several limitations constrain its broader application. The procedure requires access to high-resolution flow cytometry platforms, strict temperature control at 4 °C to preserve mitochondrial integrity and function, and a prolonged protocol exceeding 4 h. Given the necessity for rapid isolation to maintain mitochondrial viability, these technical and logistical challenges render flow cytometry less feasible for routine or clinical applications in the MT field, unless streamlined protocols and cost-effective instrumentation become available [122].

Viability and structural integrity of mitochondria are critical determinants of success in MT. Studies have shown that fragmented mitochondria, particularly those with damaged outer membranes or disrupted cristae, can release damage-associated molecular patterns (DAMPs) triggering inflammation [123]. Therefore, techniques that preserve mitochondrial integrity during isolation are essential for safe and effective MT. Most MT protocols rely on mechanical disruption or reagent-based lysis to release cellular contents, followed by differential centrifugation to obtain crude mitochondrial fractions. However, the choice of isolation method can be cell-type dependent. A comparative study using Chinese hamster ovary cells evaluated four isolation techniques—Dounce homogenization, ultrasound, digitonin, and electroporation, and found ultrasound yielded the highest number of intact mitochondria, though purity data were lacking [124].

Post-isolation quality control is indispensable to ensure mitochondrial suitability for transplantation. Key assessments include **purity** assessed by Western blot analysis for mitochondrial markers [125,126] **functionality** assessed via Seahorse bioenergetics assay and ATP quantification [127,128]; **membrane integrity** evaluated by membrane potential dye assays; and **structural evaluation** using Electron microscopy (EM) to assess cristae density and morphology [129]. EM provides critical insights into cristae architecture, which directly influences ATP synthesis rates. Despite its value, EM is underutilized due to cost and accessibility. Importantly, membrane potential assays should not be used as surrogate markers for mitochondrial health, as cristae disruption can skew results and lead to misinterpretation [130].

Variability in isolation protocols across studies inevitably leads to differences in mitochondrial quality. In ocular MT studies, homogenization remains the predominant isolation method regardless of cell source (Table 1). However, discrepancies in post-isolation quality assessment, particularly regarding scant data on mitochondrial functionality, complicate interpretation of therapeutic outcomes [108,109].

Currently, the proportion of intact versus “non-perfect” mitochondria used in MT remains undefined. Nevertheless, studies report successful rescue of diseased cells, raising questions about whether therapeutic benefit arises from functional mitochondria alone or a combination of partially damaged organelles and minor contaminants, predominantly in retinal disease models. For translational applications of MT in Ophthalmology, rigorous and standardized quality control of isolated mitochondria is imperative to ensure reproducibility and clinical relevance.

##### 1.5.2.2 Optimal source of mitochondria for ocular transplantation

Mitochondria are increasingly recognized not only as the cell's powerhouse but also as central regulators of diverse cellular processes and hence represent a promising therapeutic target across a spectrum of diseases. These organelles are highly dynamic and responsive to environmental cues. Studies in microalgae [131] and stem cells [132] have demonstrated that mitochondrial morphology and function adapt in response to changes in cellular context. Moreover, distinct mitochondrial subpopulations with unique molecular signatures have been identified within single cell types, suggesting functional heterogeneity and specialization. This raises the intriguing possibility that transplanted mitochondria may co-evolve or integrate functionally with host cells over time. Recent findings further reveal that mitochondria derived from different tissues exhibit unique pharmacodynamic profiles and substrate preferences for energy metabolism [127]. Such tissue-specific metabolic traits underscore the importance of donor-recipient compatibility in MT. Selecting an appropriate cell source for mitochondrial isolation is therefore critical, as donor mitochondria must be metabolically and functionally aligned with the host cell environment to ensure therapeutic efficacy. Encouragingly, both allogeneic and heterologous MT have been shown to be well tolerated [133], supporting the feasibility of cross-tissue or cross-individual transplantation strategies. However, optimizing donor selection based on tissue origin and metabolic compatibility remains a key consideration for advancing MT toward clinical application. In this section, we discuss the factors pertaining to this matter:

***i. Functional compatibility****:* Functional compatibility between donor mitochondria and host cells is a critical determinant of long-term therapeutic efficacy, particularly in ocular tissues. Mitochondria are multifaceted organelles involved in cellular homeostasis, including regulation of inflammation, apoptosis signaling [134] and response to ROS [135]. Emerging evidence suggests that mitochondrial behaviour varies significantly across cell types [136,137]. For instance, mitochondria from RPE cells preferentially utilize proline as a metabolic substrate, supporting antioxidant production and protein synthesis in the retina [138,139]. Moreover, ocular mitochondria are uniquely adapted to chronic oxidative stress from light exposure and high metabolic demand [140]. Mitochondria from differentiated cells like RPE exhibit higher oxidative capacity and mature cristae architecture compared to those from undifferentiated stem cells, which are more glycolytic and structurally immature [141]. These differences may influence the extent of functional rescue conferred to recipient cells following transplantation.

Recent retinal studies have explored MSCs as a source of donor mitochondria. Originally used for cell-based therapies, MSCs were found to transfer mitochondria to compromised cells [142]. Despite their immature cristae and low oxidative capacity, mitochondria from MSCs have demonstrated therapeutic benefits in ocular models. For example, mitochondria isolated from umbilical cord-derived MSCs transplanted into RPE cells reduced aging markers (β-galactosidase activity, p21, p16) via mitophagy [108]. Moreover, the application of MT to oligomeric amyloid-beta (oAβ) *in vitro* AMD model led to reduced cellular ROS, promoted ATP production and restored ZO-1 protein [111]. Further studies compared mitochondria from human Wharton's Jelly-derived MSCs (hWJ-MSCs-mt) and human endometrium-derived MSCs (hE-MSCs-mt) in AMD RPE model. Interestingly, hWJ-MSCs-mt restored mitophagy-related proteins, while hE-MSCs-mt enhanced RPE-specific protein expression, suggesting that donor cell origin influences the type of cellular response [143]. Bioenergetic outcomes post-transplantation were not reported.

Similar findings have emerged in cardiac models, where mitochondria from four different cell types (cancer cells, fibroblasts, muscle cells, cardiomyocytes) were transplanted into doxorubicin-treated cardiomyocytes. Donor-host cell type matching yielded superior rescue effects, underscoring the importance of metabolic compatibility [133]. To date, no studies have investigated autologous mitochondrial transplantation in ocular disease.

In cornea-focused studies, mitochondria isolated from kidney epithelial cells were transplanted into corneal explants from patients with Fuchs endothelial corneal dystrophy, resulting in reduced apoptosis and ROS levels despite the heterologous origin [110]. Additionally, mitochondria from immortalized RPE cells (ARPE-19) enhanced proliferation in corneal epithelial wound models [109]. These findings suggest that donor mitochondria from other cell types may augment cellular function, although direct evidence of functional recovery remains limited.

***ii. Homogenic vs. xenogeneic sources****:* In MT, the source of donor mitochondria, whether homogenic (same species) or xenogeneic (different species), can significantly influence therapeutic outcomes. Comparative studies have revealed notable differences between human and mouse mitochondria, including variations in size, volume, and network complexity within intermyofibrillar compartments [144]. Additionally, mtDNA copy number differs across organs between species, which may impact mitochondrial function and integration [145]. Since many mitochondrial proteins are encoded by nuclear DNA and imported into mitochondria, xenogeneic transplantation requires donor mitochondria to rely on host nuclear machinery for protein synthesis and maintenance. This raises concerns about mito-nuclear compatibility across species. In ocular research, all *in vivo* MT studies to date have employed homogenic sources. However, xenogeneic MT has been explored in other organ systems with promising results. For example, mitochondria isolated from hamsters and transplanted into rat neurons and astrocytes following brain ischemia led to reduced infarct size one-month post-transplantation, without immune cell activation. BrdU labelling of donor mitochondria suggested persistence of hamster mtDNA in host cells [146]. Similarly, mouse mitochondria co-incubated with human p^0^ cells (lacking functional mitochondria) restored respiration, with evidence of mitochondrial internalization [147].

Expanding on this concept, a recent study evaluated autologous, allogeneic, and xenogeneic (mouse-derived) mitochondria in an ex vivo porcine lung transplantation model [148]. All donor types preserved lung function, measured by P/F ratio and peak airway pressure, without triggering inflammation or morphological damage within 4 h post-transplantation. While these findings support the feasibility of xenogeneic MT, they reflect acute responses in ischemia-reperfusion injury models. Long-term efficacy and cross-species mito-nuclear compatibility remain uncertain.

In conclusion, mitochondria from allogeneic donors have demonstrated therapeutic potential in ocular tissues and disease models. However, xenogeneic MT warrants further investigation in the eye to assess its ability to target specific disease phenotypes. Additional research is needed to understand how the diseased host environment influences the survival, integration, and function of transplanted mitochondria.

##### 1.5.2.3 Delivery of mitochondria in ocular tissues

MT can be achieved via direct injection, intravenous infusion, or even oral administration [149]. *In vitro* and *in vivo* internalization of isolated mitochondria is typically assessed using two main approaches: detection of donor mtDNA in host cells via polymerase chain reaction (PCR) [107] or fluorescent tagging of mitochondria to visualize their behaviour. Fluorescent labelling allows dynamic observation of mitochondrial localization, fusion with host mitochondria [150], and time-lapse tracking [151], providing insights into uptake and persistence. Flow cytometry is commonly used to quantify mitochondrial internalization.

The eye's anatomical accessibility makes it a favourable site for MT. For anterior segment applications, such as corneal disorders, mitochondria are often delivered as eye drops (25 μL per drop), offering a minimally invasive approach [110]. In a mouse model of alkali-induced corneal injury, six eye drops administered every 30 min over four days promoted corneal regeneration, although the mitochondrial concentration was not specified. For posterior segment diseases involving the retina, RPE, and optic nerve, such as LHON or AMD, intravitreal injection is the preferred delivery method [111,112]. While robust evidence of mitochondrial uptake in the retina remains limited, co-incubation of isolated mouse liver mitochondria with oxidatively stressed retinal precursor cells (661W) showed preferential internalization and survival of donor mitochondria for up to 72 h [107]. In patient-derived corneal explants, cells with higher endogenous mitochondrial mass internalized more exogenous mitochondria [109]. Xenogeneic MT has also been explored in ocular tissues. Using a human-specific mitochondrial marker, transplanted human mitochondria were detected in multiple retinal layers including the ganglion cell layer, inner and outer nuclear layers, and RPE [111]. Uptake in the RPE appeared minimal compared to other layers, suggesting MT may be more effective for optic neuropathies than RPE-related disorders. To enhance mitochondrial uptake, strategies such as surface modification with cell-penetrating peptides and nanoparticle incorporation [112] have been investigated. These approaches may help overcome tissue-specific barriers in ocular MT.

Despite limited internalization, mitochondrial uptake in retinal cells has been sufficient to promote cellular rescue. However, key questions remain regarding the duration of donor mitochondrial persistence and the potential need for repeated MT. Most studies report significant metabolic recovery with only a small fraction of cells internalizing mitochondria. For example, ∼20 % of corneal cells internalized mitochondria [110], a higher rate than ischemic cardiac myocytes (3–7 %) [152], brain cells (9 %) [146], and neurons (>20 %) [153]. Hence, the number of mitochondria required for therapeutic effect varies by tissue. Feeding similar amount of isolated mitochondria for different cell types may lead to excessive uptake, causing ROS overproduction. An additional point is that variability in uptake across studies may stem from differences in quantification methods (e.g., BCA assay vs. particle count), delivery mode (contact vs. non-contact), and donor cell source.

Taken together, these findings suggest that each tissue has a threshold for mitochondrial internalization, likely regulated by mitochondrial dynamics, biogenesis, and ROS levels. Recent evidence suggests that therapeutic benefit from MT may not require long-term engraftment. Internalized mitochondria can be degraded via mitophagy after initiating beneficial effects such as mitochondrial biogenesis [154]. Further mechanistic studies in ocular tissues are needed to clarify these dynamics.

The eye's immune-privileged status enhances its suitability for MT. Inflammatory responses are dampened by immunosuppressive molecules, allowing tolerance to higher immune activation [155]. To date, only one study has assessed *in vivo* biocompatibility of isolated mitochondria in the eye. In a LHON mouse model, mitochondria modified with mitophagy-promoting nanoparticles were injected intravitreally. Mitochondria isolated from mouse heart showed no significant elevation in inflammatory cytokines (TNF-α, IL-1β, IL-6, IFN-γ) one-week post-injection across all doses [112]. However, cytokine responses at earlier time points remain unexplored.

##### 1.5.2.4 Efficacy of Mitochondrial Transplantation in eye disorders

Over the past two decades, numerous *in vitro* and *in vivo* studies have demonstrated that transplanted mitochondria via contact-independent MT can promote metabolic and phenotypic recovery across various disease models—including cardiac, neurological, and cancer. Reported benefits include anti-inflammatory effects, restoration of mitochondrial dynamics, attenuation of oxidative stress, and functional improvement. These therapeutic mechanisms are highly relevant to ocular diseases, which often share similar pathophysiological features.

In corneal disorders, endogenous mitochondrial transfer between cells has been observed *in vivo* under stress conditions, contributing to wound healing. This natural phenomenon has inspired the use of MT as a therapeutic strategy to enhance corneal epithelial repair. In optic neuropathies, MT may reduce mitochondrial mutational load and improve cellular bioenergetics, potentially alleviating symptoms and restoring visual function. For RPE degeneration (seen in diseases like AMD and DR) MT offers a unique advantage by directly addressing intrinsic mitochondrial dysfunction, which is not targeted by conventional pharmacological therapies. The metabolic remodelling induced by MT may revive compromised RPE cells and promote tissue regeneration (Fig. 1).

To rigorously evaluate MT efficacy in ocular diseases, the use of appropriate *in vitro* and *in vivo* models is essential. *In vitro* systems offer flexibility for controlled experimentation, allowing researchers to visualize donor mitochondria behaviour using techniques such as time-lapse fluorescence microscopy. These models facilitate detailed mechanistic studies in accessible and reproducible environments. In contrast, *in vivo* models are indispensable for establishing proof-of-concept and assessing functional rescue, immune responses, and biodistribution of transplanted mitochondria which are critical parameters for clinical translation. Ideally, disease models should exhibit both mitochondrial dysfunction and relevant phenotypic manifestations. This dual expression enables researchers to assess how restoring mitochondrial function via MT can reverse disease features and improve cellular health.

Currently, MT in ocular diseases is still in the nascent stage. Nonetheless, emerging data from *in vitro* studies are promising (Table 1). For example, injection of isolated human mitochondria has been shown to exert anti-inflammatory effects, rescue human umbilical vein endothelial cells and mesenchymal stem cells damaged by H_2_O_2_-induced oxidative stress, and reduce mutational load through activation of mitochondrial biogenesis [108]. These findings underscore the therapeutic potential of MT and highlight the importance of continued exploration using disease-relevant ocular models.

In this section, we discuss suitable ocular disease models and the observed benefits of MT, with a focus on its translational promise in ocular metabolic disorders.

a) Corneal diseases

Mitochondrial dysfunction has been implicated in several corneal epithelial disorders, including dry eye disease, neurotrophic keratopathy, and diabetic keratopathy, all of which are characterized by impaired epithelial integrity, delayed wound healing, and elevated oxidative stress. To evaluate the therapeutic potential of MT in these contexts, both *in vitro* and *in vivo* functional assays have been employed.

Diseased ***in vitro* models** consistently exhibit reduced mitochondrial function [112], disrupted mitochondrial morphology [15], and increased cellular ROS levels [39]. Functional assays such as the scratch assay are commonly used to assess wound healing capacity, while tight junction integrity, critical for maintaining corneal barrier function, is evaluated through junctional protein expression [156].

***In vivo***, sodium fluorescein staining enables non-invasive assessment of epithelial damage and tight junction disruption [157], whereas haematoxylin and eosin staining provides histological insights into epithelial loss, fibrosis, inflammation, vascular changes, and stromal thinning [158].

**MT efficacy** has been demonstrated in ex vivo explants from patients with late-stage Fuchs endothelial corneal dystrophy, where co-incubation with HEK293T-derived mitochondria for 48 h significantly reduced cellular ROS, likely due to elevated antioxidant content in donor mitochondria [110]. Mitophagy, assessed via Lysotracker, was also decreased, suggesting stabilization of mitochondrial turnover. In a murine model of alkali-induced corneal injury, administration of liver-derived mitochondria promoted epithelial wound healing without inducing abnormal stromal morphology, further supporting the regenerative potential of MT in corneal epithelial repair.

b) Optic neuropathies

i. Glaucoma

Mitochondrial dysfunction is increasingly recognized as a key contributor to RGC degeneration in glaucoma. Both *in vitro* and *in vivo* models have been developed to recapitulate mitochondrial pathology and evaluate the therapeutic potential of MT.

***In vitro* glaucoma models** commonly employ hypoxia [41] and glucose deprivation [159] to induce mitochondrial stress in R28 cells, a retinal precursor cell line. These conditions lead to pronounced mitochondrial damage, evidenced by increased cell death, reduced mitochondrial membrane potential, elevated mitochondrial fragmentation, and heightened ROS production. These phenotypes closely mirror the mitochondrial dysfunction observed in glaucomatous neurodegeneration, making them suitable platforms for mechanistic studies and therapeutic screening.

***In vivo* models of glaucoma** typically involve elevation of intraocular pressure [160] to simulate disease pathology. One such model induces acute ocular hypertension by raising IOP to 120 mmHg for 45 min, resulting in disintegration of mitochondrial cristae, increased cellular ROS, and RGC death [41]. The DBA/2J (D2) mouse model is also widely used to study chronic glaucoma. These mice develop age-related glaucoma characterized by shortened and fragmented mitochondria within RGC axons, which also exhibit reduced axonal length [161]. Collectively, these models demonstrate well-characterized mitochondrial dysfunction and provide robust platforms for evaluating MT efficacy. To enhance translational relevance, future studies should incorporate additional functional readouts such as electrophysiology to assess the extent of neuroprotection and functional rescue following MT.

**Evidence of MT efficacy**: Initial studies have demonstrated the therapeutic potential of MT in promoting RGC axonal regeneration using an optic nerve crush model [104]. In this model, rat optic nerves were crushed for 15 s, followed by intravitreal injection of isolated rat liver mitochondria. MT led to significant axonal regeneration, evident by day 28 post-injury. Notably, transplanted mitochondria improved oxidative metabolism and electrophysiological responses as early as one day after administration, underscoring the rapid and functional benefits of MT in glaucomatous injury.

ii. Hereditary Optic Neuropathies - LHON models and MT applications - ADOA models and MT considerations

Hereditary optic neuropathies, including Leber's Hereditary Optic Neuropathy (LHON) and Autosomal Dominant Optic Atrophy (ADOA), are characterized by mutations in mitochondrial protein-coding genes that impair mitochondrial function and lead to degeneration of RGCs. These conditions are ideal candidates for MT, given their direct link to mitochondrial dysfunction.

LHON models and MT applications

LHON is commonly modelled using patient-derived fibroblasts and cybrid systems harbouring mutations such as m.3460G > A (MT-ND1) and m.11778G > A (MT-ND4). These cells exhibit elevated mitochondrial and cellular ROS, reduced autophagy and mitophagy, and other hallmarks of mitochondrial stress [162]. Importantly, LHON patients fall into two categories: carriers and affected individuals. Carriers often exhibit compensatory mechanisms that prevent cell death, which must be considered when evaluating MT efficacy [163].

To better model disease-relevant cell types, RGCs should be used, as they are the primary site of injury. ***In vitro* models** have been successfully generated using iPSC-derived RGCs from a LHON-affected family. These cells displayed altered soma and neurite morphology, impaired electrophysiological responses, reduced ATP levels, and elevated caspase-3 activity [164]. This model, which incorporates patient-specific mutations and mitochondrial pathology, is well-suited for MT investigation.

***In vivo,*** LHON is commonly modelled by intravitreal injection of rotenone, a complex I inhibitor. This induces mitochondrial cristae loss, reduced mitochondrial number and volume [165] in the inner plexiform and ganglion cell layers, decreased retinal thickness, and attenuated electroretinogram responses [166]. More recently, transgenic mice carrying the human ND6 G14600A (P25L) [167] LHON mutation have been developed, offering a genetically relevant platform for MT studies.

MT has been applied in LHON models targeting complex I dysfunction. One study utilized nanoparticles loaded with PARKIN mRNA to modify isolated mouse heart mitochondria [112], promoting mitophagy and acute rescue of diseased RGCs. Following MT, complex I expression and activity were restored, with reductions in mitochondrial ROS and membrane potential. Rotenone-injected mice showed improved ATP levels, retinal morphology, and thickness. Behavioural assays revealed enhanced optomotor responses, with MT-treated mice displaying head movement counts comparable to controls, underscoring the therapeutic promise of MT in LHON. Future studies can investigate mtDNA heteroplasmic changes in LHON after MT.

ADOA models and MT considerations

ADOA is primarily caused by mutations in the OPA1 gene, which disrupt mitochondrial dynamics and bioenergetics [168]. Patient-derived cells exhibit reduced mitochondrial function, mtDNA defects, and increased apoptosis markers. Lymphoblastoid cells from ADOA patients show fragmented mitochondria, decreased mtDNA copy number, reduced membrane potential, and diminished expression of mitochondrial proteins [169]. Although RGC-specific models are preferred, these cell lines provide useful proof-of-concept platforms for MT.

***In vivo***, an RGC-specific *Opa1* knockout mouse model has been developed. Behavioural assays, including optokinetic and forced-swimming tests, revealed diminished visual responses. While mitochondrial dysfunction was not extensively characterized *in vivo*, *in vitro* data confirmed reduced mitochondrial mass [170].

Given the persistent nuclear mutation in ADOA, MT is unlikely to correct the underlying genetic defect. However, it may alleviate ocular symptoms and improve cellular health. MT could serve as an adjuvant to existing therapies, potentially offering synergistic and sustained benefits.

Current studies demonstrate encouraging advances in applying MT to hereditary optic neuropathies. Critical questions remain regarding the durability of therapeutic effects, survival of transplanted mitochondria within host cells, and the duration of metabolic rescue following a single MT dose. As the field evolves, it is essential to determine whether MT can not only restore mitochondrial health but also modify disease phenotypes in a meaningful and lasting way.

c) Retinal Disorders

i. Age related macular degeneration (AMD)

The development of robust experimental models is critical for evaluating the therapeutic efficacy of MT in AMD. Both *in vitro* and *in vivo* systems have been employed to recapitulate key pathological features of AMD, particularly those associated with mitochondrial dysfunction.

***In vitro* models:** Patient iPSC derived retinal pigment epithelium cells exposed to oxidative stressors such as hydrogen peroxide have been widely utilized to model AMD-associated mitochondrial dysfunction. Even without exposure to stress, patient iPSCs exhibit hallmark features including reduced antioxidant response, disrupted mitochondrial cristae, impaired biogenesis, and lipid accumulation reminiscent of drusen formation [171]. Hydroquinone, a constituent of cigarette smoke, has also been employed to induce oxidative stress [172]. This model demonstrates abnormal mitochondrial morphology and reduced bioenergetics, with downregulated autophagy implicated as the underlying mechanism. However, phenotypic changes characteristic of AMD are not consistently observed in this system. Recent advances have led to the development of a three-dimensional human cell model comprising RPE, Bruch's membrane, and choriocapillaris [173]. Upon exposure to complement serum or hypoxia-inducible factor 1-alpha, this model can simulate dry and wet AMD phenotypes, respectively. Given its human origin and structural complexity, it offers a clinically relevant platform for investigating MT and its potential to restore mitochondrial integrity and cellular function.

***In vivo* models** provide essential insights into the physiological impact of MT. Sodium iodate-treated mice are among the most commonly used AMD models, exhibiting lipofuscin accumulation, retinal thinning, and mitochondrial cristae disruption in RPE cells. Proteomic analyses have revealed elevated expression of complement components and vitronectin, aligning with AMD pathology [174]. Another model involves repression of peroxisome proliferator-activated receptor gamma coactivator 1-alpha (PGC-1α), a master regulator of mitochondrial biogenesis. Mice with reduced PGC-1α expression develop drusen-like deposits and exhibit impaired photoreceptor and RPE function. Although mitochondrial dysfunction was not directly characterized *in vivo*, *in vitro* studies using PGC-1α knockout cells revealed reduced mitochondrial DNA copy number, compromised membrane potential, and increased susceptibility to oxidative stress [175]. CLIC4 knockout mice represent a promising model, closely mimicking AMD features such as age-associated visual decline, drusen accumulation, and transcriptomic alterations [176].

Despite their utility, murine models lack macula, an anatomical structure central to AMD pathogenesis, limiting their translational relevance. Rabbit eyes, while morphologically similar to human eyes, also lack macula but may facilitate technical adaptations for human application [177]. In contrast, non-human primates (NHPs) possess macula and share structural similarities with the human eye, making them the most suitable model for *in vivo* MT studies. Future research should prioritize NHP models to enhance clinical translatability [178].

There remains a critical need to develop and characterize models that concurrently exhibit AMD phenotypes and mitochondrial dysfunction. Such systems would enable more accurate assessment of MT's therapeutic potential and facilitate the translation of preclinical findings into clinical applications.

**Evidence of MT efficacy** has been investigated in models using oligomeric amyloid-beta (oAβ) in both *in vitro* and *in vivo* settings. In ARPE-19 cells, MT resulted in decreased mitochondrial ROS, improved membrane potential, and elevated ATP levels. Notably, expression of ZO-1, a key structural protein in RPE, was restored following MT. These effects were also observed *in vivo*, with preliminary evidence suggesting that the Akt/GSK3β signalling pathway may mediate the observed rescue [111].

### 1.6 Potential mitochondrial biomarkers and diagnostic tools (to assess efficacy in humans)

Extrapolating any treatment's efficacy requires the accurate identification, and robust measurement of biomarkers for that disease. Unfortunately, there are currently no *in-vivo* tools that track mitochondrial health directly within the ocular environment in a non-invasive fashion. In this section, we discuss various techniques and clinical tools that may be used to evaluate the efficacy of MT clinically.

#### 1.6.1 Flavoprotein fluorescence

First used in 1984 as a non-invasive means of measuring *in-vivo* redox activity of corneal mitochondria, the evolving use of flavoprotein fluorescence (FPF) has allowed it to remain as an important biomarker for the real-time assessment of dysfunctional mitochondria. Confocal microscopy and fluorescence lifetime imaging have confirmed the mitochondrial origin of FPF and its sensitivity to changes in mitochondrial membrane potential and metabolic flux [179,180]. FPF primarily originating from oxidized flavin adenine dinucleotide (FAD), serves as a sensitive optical biomarker of mitochondrial redox state and metabolic activity. Under physiological conditions, FPF intensity reflects active electron transport and OxPhos. However, in pathological states such as DR, AMD, and glaucoma, elevated FPF paradoxically indicates mitochondrial stress, uncoupling, or compensatory hyperactivity in dysfunctional cells [181]. Studies have shown that regions of increased FPF in the retina often correlate with disease severity and visual acuity loss, suggesting that FPF elevation may reflect maladaptive metabolic responses rather than improved function. Following MT, successful integration of healthy mitochondria is expected to restore redox balance and normalize FPF signals, making it a promising non-invasive readout for assessing MT efficacy in retinal models.

#### 1.6.2 Optical coherence tomography

Traditional optical coherence tomography has revolutionized retinal imaging by providing high-resolution cross-sectional views of tissue architecture. However, its utility is largely confined to structural assessment, with limited capacity to resolve subcellular features or capture dynamic physiological processes such as mitochondrial activity. The axial resolution of conventional OCT, typically in the range of 3–10 μm, precludes direct visualization of organelles like mitochondria, which are submicron in scale. This issue arises from the coherence length of the light source and the bandwidth constraints inherent to standard OCT systems [182]. While ultrahigh-resolution OCT (UHR-OCT) can approach submicron axial resolution under optimized conditions, even these systems are unable to visualize mitochondria *in vivo* [183]. Comparative studies using visible-light OCT and electron microscopy confirm that subcellular features such as the ellipsoid zone and RPE organelle distribution can be inferred but not directly visualized [184]. To address these limitations, emerging functional OCT techniques have been developed to probe retinal bioenergetics and cellular physiology *in vivo*. Stimulus-evoked OCT is a novel way to assess mitochondrial respiration *in vivo* by capturing dynamic changes within the PR-RPE support network [185]. This network plays a critical role in maintaining retinal homeostasis, particularly within the subretinal space (SRS), where pro-survival signalling is tightly regulated by mitochondrial activity. Stimulus-evoked OCT can detect transient optical changes in the SRS that reflect shifts in hydration and tissue reflectivity—both of which are influenced by mitochondrial respiration in photoreceptors. These changes serve as indirect markers of bioenergetic flux and can be used to evaluate the functional integrity of the photoreceptor–RPE axis. By monitoring these responses, OCT may help identify early mitochondrial dysfunction, guide therapeutic interventions, and track the efficacy of treatments aimed at restoring metabolic resilience in retinal diseases. Phase-sensitive [186] and polarization-sensitive OCT [187] variants further enhance contrast by capturing subtle changes in tissue birefringence and scattering, which may reflect mitochondrial integrity and cytoskeletal dynamics. These functional extensions of OCT are increasingly being integrated with complementary modalities such as fluorescence imaging and adaptive optics [188] enabling a more comprehensive evaluation of retinal health. Collectively, these advances position functional OCT as a promising tool for detecting early bioenergetic deficits and monitoring therapeutic response in mitochondrial retinal diseases.

Fluorescence lifetime imaging ophthalmoscopy (FLIO) detects metabolic changes in tissues as autofluorescence lifetimes. These are typically described as the fluorescence decay over time without the application of fluorescent dye and are distinctive to various metabolic alterations specific to various diseases. FLIO-OCT is being used to evaluate retinal and RPE health based on their metabolic changes in response to their microenvironment [189].

#### 1.6.3 Mitochondrial DNA as a biomarker for clinical diagnosis

mtDNA maybe investigated as a potential biomarker for evaluating MT in ocular diseases associated with bioenergetic dysfunction [190,191]. Although its clinical utility remains to be validated, mtDNA quantification may offer insights into the persistence and integration of transplanted mitochondria, while assessments of mtDNA integrity and mutation burden could reflect their functional viability. Emerging evidence also suggests that mtDNA may serve as a systemic indicator of retinal stress. For instance, a recent case-control study reported elevated serum mtDNA levels in treatment-naïve patients with acute central serous chorioretinopathy, with concentrations correlating with the height of serous retinal detachment [192]. These findings, while preliminary, highlight the potential relevance of mtDNA in capturing mitochondrial perturbations in retinal disease.

Nonetheless, mtDNA analysis has notable limitations. Quantitative measures alone may not reflect functional competence or integration into host bioenergetic networks [193]. Peripheral sampling may not accurately represent retinal mitochondrial status, and assay variability combined with the absence of standardized thresholds limits clinical translation [194]. Longitudinal studies are needed to determine whether mtDNA dynamics correlate with therapeutic response or disease progression, and patient stratification based on baseline mitochondrial profiles may further refine its utility. Therefore, mtDNA is best utilized as part of a multimodal assessment strategy, integrated with metabolic imaging and functional assays to comprehensively evaluate transplantation success.

#### 1.6.4 Metabolomics as a source of biomarkers

Metabolomics is the comprehensive study of small molecule metabolites that participate in cellular metabolism. These molecules are highly sensitive to disease progression, therapeutic interventions, and environmental influences, making them valuable indicators of biological change [195,196]. In ocular diseases such as AMD, glaucoma, DR, and myopia, specific metabolic pathways including purine, taurine, glutamate, and lipid metabolism have been consistently implicated [197]. Recent studies have classified AMD into distinct metabolomic profiles, each associated with unique mitochondrial signatures and disease stages [198,199]. These include changes in beta-citrylglutamate, kynurenate, acyl carnitine, and sphingomyelins, which reflect disruptions in energy metabolism and mitochondrial function [200,201].

Following mitochondrial transplantation, metabolomics offers a powerful tool to evaluate therapeutic impact beyond structural or genetic markers. Evidence from non-ocular models such as cardiac injury, neurodegeneration, and cancer shows that transplanted mitochondria can reshape host metabolite profiles, restore energy pathways, and suppress disease-related signalling [202]. These shifts include recovery of fatty acid oxidation intermediates, reduction of glycolytic and tumour-associated metabolites, and reprogramming of immune cell metabolism. Although ocular-specific data are limited, these findings highlight the importance of metabolomic profiling in mitochondrial therapy research.

Donor age and mitochondrial phenotype may further influence metabolite outcomes. Studies have shown that younger mitochondria can more effectively reduce glucose and lactate levels, disrupt aerobic glycolysis, and suppress cancer-related metabolites [203]. As mitochondrial therapies evolve, metabolomics will be essential for monitoring treatment response, guiding donor selection, and uncovering mechanistic insights that support precision medicine.

### 1.7 Future directions and challenges

**Fig. 2:**
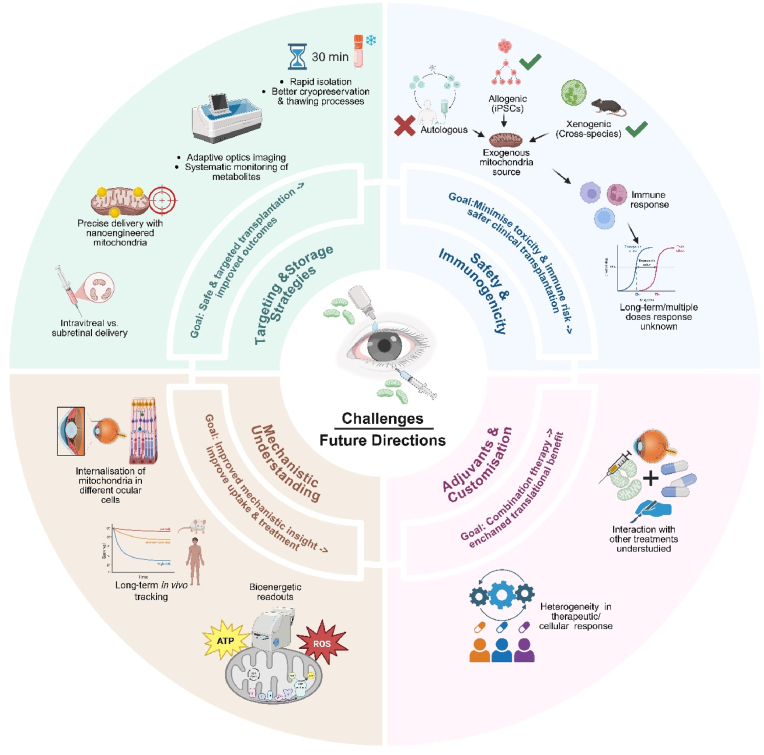
**Overview of Challenges and Future Directions in Mitochondrial Transplantation (MT) for Ocular Diseases.** The figure represents the most significant challenges faced during MT for ocular mitochondrial dysfunction (1) Safety & Immunogenicity: highlighting the need to minimize immune responses via optimizing donor compatibility and long-term tracer studies; (2) Targeting & Storage: focusing on precision of mitochondrial delivery at subcellular level augmented with imaging tools and optimization of mitochondrial cryopreservation techniques; (3) Mechanistic Understanding: emphasizing the need to elucidate mechanistic underpinnings of mitochondrial internalization, bioenergetic impact, and *in vivo* tracking; and (4) Adjuvants and Customisation: addressing the development of robust *in vitro* systems and understanding cellular heterogeneity. These themes converge on the central challenge of translating MT into safe, scalable, and effective ocular therapy.

Mitochondrial transplantation is a rapidly evolving therapeutic strategy aimed at alleviating mitochondrial dysfunction in ocular diseases. While significant progress has been made in preclinical models, several critical challenges must be addressed to enable successful clinical translation. Overcoming these barriers will facilitate the transition of MT from bench to bedside (Fig. 2).

#### 1.7.1 Overcoming delivery and targeting challenges

Despite promising *in vitro* outcomes, the greatest challenge in ocular MT lies in achieving precise subcellular delivery *in vivo*. The eye comprises diverse cell types, and diseases such as AMD affect multiple layers, most notably the RPE and PRs. This necessitates careful selection of delivery routes, such as subretinal or intravitreal injections. Although both are invasive, intravitreal injection is generally preferred due to its procedural simplicity, reduced risk of retinal detachment, and avoidance of bleb induction [204]. Although universal distribution of mitochondria within all ocular tissues via intravitreal injection may be beneficial, mitochondrial transit to the most posteriorly located RPE may not be as robust as that for RGCs or neural retina. It remains unclear whether excess mitochondrial accumulation in unaffected tissues could trigger inflammation or immune responses. Therefore, it is imperative to develop clinically optimized delivery strategies targeting specific cell types - RGCs, RPE, and PRs.

While direct injection and other enhancement procedures using extra physical force e.g. Mito-ception [205], MitoPunch [206], Photothermal blade [202], optical tweezers or microfluidics may be relevant to other organs like heart, muscle, lung or kidneys, they are inapplicable in eye field due to the risk of damage to the fragile ocular tissues. Though metabolically dysfunctional tissues are reported to have higher uptake of healthy mitochondria, innovative techniques aimed to selectively deliver isolated mitochondria to the dysfunctional target cells may need to be developed. This includes designing mitochondria with surface modifications such as cell penetrating peptides, polymer coatings, or ligand attachments. These features can help the mitochondria bind to specific receptors in diseased RPE, PRs, or RGCs, and improve their uptake into target cells. For example, diseased cells in advanced AMD are associated with increased expression of lipids [207] and low-density lipoprotein receptors, which could be harnessed for targeted mitochondrial delivery. However, these technological advances warrant research *in vivo* with regards to eye disorders.

Another critical consideration is the determination of the therapeutic window for MT. Early intervention, particularly during the neovascular phase of AMD when RPE cells are viable, may yield better outcomes than late-stage administration when RPE and PRs are atrophied and scarred with little to no potential for rejuvenation. This will require robust protocols to ascertain the clinical threshold to initiate MT. Assessing these clinical parameters requires precise diagnostics such as adaptive optics for high-resolution visualization of photoreceptors and RPE, along with systemic metabolomic profiling. These standard procedures can be subsequently employed to generate readouts for monitoring patients after MT.

Mitochondrial isolation protocols also present a translational bottleneck. Conventional methods require up to 2 h on ice to preserve viability. It has been demonstrated that mitochondria with superior functionality demonstrate greater therapeutic benefits, which underscores the need to develop a rapid and robust isolation protocol. A quicker isolation protocol has been developed where mitochondrial isolation from tissues can be completed in 30 min [208]. This procedure is based on utilizing differential filtration instead of multiple centrifugations, which can compromise mitochondrial quality. Minimal damage and contamination of isolated mitochondria have been demonstrated using this protocol and is in use by various research groups. These are significant advantages in the context of clinical application as short processing time can lead to better viability and functionality of isolated mitochondria. This protocol optimization coupled with cryopreservation can potentially make clinical translation a possibility. However, cryopreservation of mitochondria is a hurdle that is being tackled. A particular buffer has been shown to preserve mitochondrial structure and functions for a year when stored at −80^0^C [209]. Succinct buffers crucial for short and long-term storage, which can maintain mitochondrial functionality during transit to the clinic or is readily available after thawing the cryopreserved product, are the need of the hour. Even so, the success of cryopreservation will ultimately depend on optimizing other parameters such as thawing and freezing process and storage times.

Thus, overcoming existing barriers to delivering healthy mitochondria to target tissues will advance the clinical development of MT.

#### 1.7.2 Addressing potential side effects and toxicity

Though mitochondria are known to possess distinctive non-immunogenic properties due to their endosymbiotic nature, the possibility of adverse effects and toxicity after MT cannot be totally excluded. These risks are closely tied to the source of donor mitochondria. Thus, autologous and homogenic sources are generally preferred to minimize immune activation. While autologous MT may be feasible for localized mitochondrial dysfunction (e.g., ischemic injury in heart or kidney), it is impractical for primary mitochondrial diseases such as LHON, which involve systemic mutations. Similarly, diseases like glaucoma and AMD may have genetic predispositions affecting all cells in the body. Consequently, *in vivo* ocular MT studies have adhered to heterologous and homogenic sources. Allogeneic sources, particularly mesenchymal stem cells (MSCs), are widely used *in vitro* due to their ease of culture and favourable immunological profile. Although xenogeneic MT has been extensively explored for MT in other organs [210], it warrants extensive research in ocular tissues due to ethical implications and potential immune response. Importantly, most studies have assessed immune activation only after a single MT dose and over short durations. It remains unknown whether repeated dosing, likely necessary in clinical settings, could elicit immune responses. Induced pluripotent stem cells (iPSCs) and MSCs represent promising sources for generating exogenous mitochondria, potentially expanding the scope of allogeneic MT.

#### 1.7.3 Understanding mechanism of mitochondrial rescue

The cellular heterogeneity of ocular tissues influences the mechanism of mitochondrial uptake. RPE cells, being inherently phagocytic, may internalize mitochondria via phagocytosis, whereas retinal neurons and RGCs likely rely on endocytosis. Current studies lack detailed mechanistic insights into MT across different ocular cell types. Understanding these pathways is essential for optimizing uptake and enhancing therapeutic efficacy.

Moreover, it remains unclear whether long-term engraftment of transplanted mitochondria is necessary for sustained benefit, or if transient improvements in homeostasis such as mitophagy activation and degradation of donor mitochondria are sufficient. Long-term tracer studies are needed to track mitochondrial fate *in vivo* and determine optimal dosing intervals. Future research should also prioritize bioenergetic assessments post-MT, which are currently underreported, to complement phenotypic rescue data.

#### 1.7.4 Combining MT with existing therapies

MT may offer enhanced therapeutic outcomes when used in combination with existing treatments. Ocular diseases are often multifactorial, and targeting multiple pathways may yield superior results. For example, in wet AMD anti-VEGF therapy effectively suppresses choroidal neovascularization but may contribute to RPE and PR atrophy with repeated dosing. MT could complement anti-VEGF therapy by rejuvenating metabolically compromised RPE and PR cells. Such combinatorial approaches must be tailored to specific ocular cell types, as cellular heterogeneity influences MT responsiveness. Additionally, interactions between MT and other therapies must be systematically evaluated to ensure safety and efficacy.

## 2 Conclusion

Mitochondrial transplantation has demonstrated therapeutic potential across various ocular tissues affected by mitochondrial dysfunction. Reported benefits include regeneration of corneal epithelial cells, enhancement of cellular bioenergetics, axonal elongation and restoration of mitochondrial homeostasis in retinal ganglion cells (RGCs), and phenotypic rescue with attenuation of oxidative stress in retinal pigment epithelium cells. These findings underscore the promise of MT as a novel intervention for chronic and degenerative eye diseases.

However, current outcomes must be substantiated by more rigorous data, particularly regarding post-transplantation bioenergetics and the molecular mechanisms underlying mitochondrial rescue. Additionally, the optimal source of donor mitochondria remains to be defined, as it may influence therapeutic efficacy and immunological compatibility. A deeper understanding of these parameters will be instrumental in addressing key challenges, including targeted delivery to specific ocular cell types and mitigating potential adverse effects.

Future research should prioritize longitudinal studies in appropriate animal models to evaluate the durability and safety of repeated MT. These investigations are essential prerequisites for advancing MT toward clinical trials and eventual therapeutic application.

In summary, MT represents a rapidly advancing therapeutic modality in ophthalmology. Bridging current knowledge gaps particularly in mitochondrial targeting, mechanistic elucidation, and long-term fate of transplanted mitochondria will be pivotal in unlocking its full clinical potential. With continued innovation and translational research, MT may redefine the management of chronic and debilitating ocular disorders, offering a powerful tool for restoring vision and cellular health.

## Declaration of generative AI and AI-assisted technologies in the writing process

During the preparation of this work the author(s) used Microsoft co-pilot in order to improve language and readability only. After using this tool/service, the author(s) reviewed and edited the content as needed and take(s) full responsibility for the content of the publication.

## Funding

This work was supported by the National Medical Research Council (NMRC): New Investigator Grant (NMRC–CS–IRG-NIG, Grant no: MOH-001076-00) and NUHS Clinician Scientist Programme (NCSP, Grant no: NSCP2.0/2024/NUHS/MB). It is also supported by National University Hospital: NUH SEED Fund (Grant no: NUHSRO/2025/005/RO5+5/Seed-Sept24/01).

## CRediT authorship contribution statement

**Yan Zhuang Yeo:** Conceptualization, Data curation, Methodology, Writing – original draft, Writing – review & editing. **Mayuri Bhargava:** Conceptualization, Data curation, Funding acquisition, Methodology, Project administration, Supervision, Writing – original draft, Writing – review & editing. **Rajvinder Singh Khare:** Conceptualization, Data curation, Writing – original draft, Writing – review & editing. **Bhav Harshad Parikh:** Conceptualization, Methodology, Supervision, Writing – review & editing. **Xinyi Su:** Conceptualization, Supervision, Writing – review & editing.

## Declaration of competing interest

The authors declare that they have no known competing financial interests or personal relationships that could have appeared to influence the work reported in this paper.

## Data Availability

No data was used for the research described in the article.
